# Comment on “Association of mean platelet volume with echocardiographic findings in patients with severe rheumatic mitral stenosis”

**DOI:** 10.15171/jcvtr.2019.55

**Published:** 2019-11-04

**Authors:** Erkan Coban

**Affiliations:** Akdeniz University, Faculty of Medicine, Department of Internal Medicine, Antalya, Turkey

I have read with great interest the recently published article by Aslanabadi et al.^1^ Authors reported that patients with rheumatic mitral stenosis has a higher mean platelet volume (MPV) compared to the healthy individuals. However, there are major limitations about MPV levels in this study;

1) In this study, MPV measurement technique is not written. The MPV is dependent on a number of variables, including time of analysis after venepuncture, method of analysis, anticoagulant used and specimen storage temperature. Pre-analytical variables, such as the anticoagulant used, and the time between blood collection and measurement are known to significantly affect MPV measurements. Although EDTA is traditionally used and recommended for samples destined for blood counting it is well known that platelets collected into EDTA anticoagulants undergo time-dependent platelet swelling and activation.^2-4^ The nature of the study leads to a significant problem because the MPV results could not be standardized. Therefore, MPV-based evaluation may give erroneous results.

## Competing interests

None.

## Ethical approval

Not applicable.
